# The influence of cardiovascular morbidity on the prognosis in prostate cancer. Experience from a 12-year nationwide Danish population-based cohort study

**DOI:** 10.1186/1471-2407-11-519

**Published:** 2011-12-15

**Authors:** Christina G Jespersen, Mette Nørgaard, Truls E Bjerklund Johansen, Mette Søgaard, Michael Borre

**Affiliations:** 1 Department of Urology, Aarhus University Hospital, Brendstrupgaardsvej 100, 8200 Aarhus N, Denmark; 2 Institute of Clinical Medicine, Aarhus University Hospital, Brendstrupgaardsvej 100, 8200 Aarhus N, Denmark; 3 Department of Clinical Epidemiology, Aarhus University Hospital, Olof Palmes Allé 43-45, 8200 Aarhus N, Denmark

## Abstract

**Background:**

To determine the impact of preexisting ischemic heart disease (IHD) and stroke on overall survival in prostate cancer patients.

**Methods:**

We conducted a cohort study of patients with incident prostate cancer registered in the Danish Cancer Registry from 1997 through 2008. We identified patients diagnosed with IHD or stroke prior to the date of prostate cancer diagnosis in the Danish National Patient Registry. We constructed Kaplan-Meier curves to analyze time to death and Cox regression was used to estimate hazard ratios (HRs) to compare mortality rates by preexisting IHD or stroke status, adjusting for age, stage, comorbidity, and calendar period.

**Results:**

Of 30,721 prostate cancer patients, 4,276 (14%) had preexisting IHD and 1,331 (4%) preexisting stroke. Crude 1- and 5-year survival rates were 85% and 44% in men without preexisting IHD or stroke, 81% and 36% in men with preexisting IHD, and 78% and 27% in men with preexisting stroke. Adjusted HRs were 1.05 (95% CI 1.00-1.10) for patients with IHD and 1.20 (95% CI 1.12-1.30) for patients with stroke compared with patients without preexisting IHD or stroke.

**Conclusions:**

Preexisting IHD had minimal impact on mortality in prostate cancer patients, whereas overall mortality was 20% higher in prostate cancer patients with preexisting stroke compared to those without IHD or stroke. These results highlight the importance of differentiating between various comorbidities.

## Background

Prostate cancer is the most common malignancy in men and the second most common cause of death from cancer in men in Western countries [1]. In Denmark, prostate cancer accounts for 4% of all deaths in men [2]. The median age of patients with newly diagnosed prostate cancer is above70 years, and a large proportion of these men have coexisting diseases (comorbidities) at the time of prostate cancer diagnosis [3]. Several studies have shown that comorbidity increases mortality in prostate cancer patients [3-6]. In a Danish population-based cohort study of prostate cancer patients diagnosed between 1995 and 2006, comorbidity was present in more than one-third of the patients and was a negative prognostic factor [3]. In that study, prostate cancer survival generally improved over time except among patients with high levels of comorbidity. However, the study did not address the impact of specific types of comorbidity. Comorbidity may indirectly affect prognosis by influencing choice of treatment; e.g. in men with localized prostate cancer those with comorbidity tend to receive conservative/nonsurgical treatment more often than those without [4,7-9]. Cardiovascular diseases, including ischemic heart disease (IHD) and stroke, are common diseases and the leading causes of death in most Western countries accounting for nearly half of all deaths in Europe (48%) [10] and of one third of all deaths in the US (34%) [11]. During the last 30 years, mortality from cardiovascular diseases has declined steadily in most European countries due to decreasing incidence combined with improved treatments [10-14]. Still, little is known on how preexisting IHD and stroke affect the survival in prostate cancer patients. The aim of this study was therefore to estimate the prognostic impact of preexisting IHD and stroke in prostate cancer patients. Accordingly, we conducted a large cohort study of Danish prostate cancer patients using nationwide registries.

## Methods

### Study population

We conducted this nationwide cohort study in Denmark, which has 5.3 million inhabitants. Free tax-supported health care is provided for all Danish residents by the National Health Service. We retrieved data from the Danish Civil Registration System, the Danish Cancer Registry, and the National Patient Registry. A unique 10-digit civil registration number is assigned to all Danish residents by the Central Office of Civil Registration, and this number allows unambiguous linkage between all Danish registries [15].

### Identifying patients with prostate cancer

During the study period (1997-2008), no formal prostate cancer screening program existed in Denmark. Men with suspected or confirmed prostate cancer were referred to departments of urology in public hospitals for examination, counselling, and treatment. We identified men with prostate cancer through the Danish Cancer Registry [16]. This is a population-based, nationwide registry with data on incident cancer in Denmark since 1943. Data include civil registration number, diagnostic measures, and stage at diagnosis. All diagnoses have been reclassified according to the International Classification of Diseases 10^th ^revision (ICD-10). We used ICD-10 code DC61.9 to identify patients with prostate cancer. Until 2003, disease stage was recorded as localized, regional, or distant. After 2003, the stage was recorded using the TNM system. We classified the stage as localized if T1-2, N0, M0, regional if T3-4 or N1-3, M0, and distant if T1-4, N0-3, M1.

### IHD and stroke

We obtained information on preexisting IHD and stroke through the National Patient Registry [17]. This registry contains data on all somatic hospital admissions since 1977 and on outpatient and emergency room visits since 1995. It includes dates of admission and discharge, surgical procedures, and up to 20 diagnoses coded by physicians at discharge according to ICD 8 until 1993 and ICD 10 thereafter. We identified all hospitalizations with primary or secondary diagnoses of IHD (angina pectoris, acute myocardial infarction, acute and chronic IHD, asymptomatic IHD), and stroke (cerebral infarction and unspecified stroke) recorded within 10 years prior to date of prostate cancer diagnosis (see Table 1 for codes).

**Table 1:** ICD-8 and ICD-10 diagnosis codes of ischemic heart disease and stroke

Diagnosis	ICD-8 codes	ICD-10 codes
Ischemic heart disease:		
Angina pectoris	413.09, 413.99	DI20.x
Acute myocardial infarction	410.09, 410.99	DI21.x, DI22.x, DI23.x
Acute ischemic heart disease	411.09, 411.99	DI24.x
Chronic ischemic heart disease	412.09, 412.99	DI25.x
Asymptomatic ischemic heart disease	414.09, 414.99	
Stroke:		
Cerebral infarction		DI63.x
Cerebral apoplexy (unspecified)		DI64.x
Cerebral thrombosis	433.09, 433.99	
Cerebral embolism	434.09, 434.99	
Cerebral apoplexy	436.01, 436.90	
Acute cerebrovascular disease	436.09, 436.99	

### Other types of comorbidity

We used the Charlson comorbidity index [18] to describe other types of comorbidity in the prostate cancer patients. We calculated the index score for each patient based on previous diagnoses other than IHD, stroke, or prostate cancer recorded in the National Patient Registry recorded within ten years before the prostate cancer diagnosis. We categorized the index score into three comorbidity levels: 0 = none, 1-2 = moderate, and ≥ 3 = high.

### Mortality

Mortality and migration updates were obtained from the Civil Registration System [15]. This system is updated daily and contains information on vital status (dead or alive), date of death, and postal address. When a Danish citizen dies, the attending physician must report the cause of death, and the events leading to death are described by up to four ICD-10 diagnoses. We obtained information on the primary cause of death for all patients from 1997 to 2008 through the Danish Cause of Death Registry, established in 1943.

### Statistical analysis

Follow-up started at the date of prostate cancer diagnosis and continued until death or December 31^st ^2008, whichever occurred first. We constructed Kaplan-Meier curves to illustrate time from prostate cancer diagnosis to death. We used Cox regression analysis to estimate hazard ratios (HRs) to compare time to death within 5 years after prostate cancer diagnosis in patients with preexisting IHD or stroke compared with prostate cancer patients without IHD or stroke, while controlling for age (categorized as 30-34, 35-39, 40-44, 45-49, 50-54, 55-59, 60-64, 65-69, 70-74, 75-79, 80-84, 85-100 years), prostate cancer stage, study year, and other comorbidities. In this analysis patients were followed until death, five years of follow-up, or December 31st 2008, whichever came first. The analysis was stratified by age group: 30-59, 60-69, and ≥ 70 years. We also restricted the analysis to patients without other chronic diseases (Charlson comorbidity level = 0). Furthermore, we estimated HRs for those diagnosed with IHD or stroke within 1 year before prostate cancer diagnosis compared with those diagnosed 2-10 years before prostate cancer diagnosis. The assumptions for the Cox model were assessed graphically and found appropriate. Estimates are presented with 95% confidence intervals (CI). Statistical analyses were performed using STATA software (Version 11, SE). The study was approved by the Danish Data Protection Agency (Journal no. 2009-41-3793).

## Results

We included 30,721 men with a primary diagnosis of prostate cancer in Denmark between 1997 and 2008. The median age at prostate cancer diagnosis was 72 years (range, 34-100 years). At diagnosis, 14% (4,276) had preexisting IHD and 4% (1,331) preexisting stroke. Patients with preexisting IHD or stroke were older, had higher tumour stage, and higher levels of other comorbidities at diagnosis than patients without IHD or stroke (Table 2).

**Table 2:** Characteristics of the 30,721 prostate cancer patients diagnosed between 1997 and 2008

Characteristics	Prostate cancer patients with IHD n (%)	Prostate cancer patients with stroke n (%)	Prostate cancer patients without cardiovascular disease n (%)	Entire cohort n (%)
No. of patients	4,276 (14)	1,331 (4)	25,114 (82)	30,721 (100)
Age at prostate cancer diagnosis, yr				
30-59	173 (4)	53 (4)	2,504 (10)	2,730 (9)
60-69	1,082 (25)	284 (21)	8,202 (33)	9,568 (31)
70-79	1,920 (45)	576 (43)	9,281 (37)	11,777 (38)
80+	1,101 (26)	418 (32)	5,127 (20)	6,646 (22)
Comorbidity level				
Score* none (0)	1,982 (46)	467 (35)	18,148 (72)	20,597 (66)
Score moderate (1-2)	1,650 (39)	647 (49)	5,791 (23)	8,088 (27)
Score high (> 2)	644 (15)	217 (16)	1,175 (5)	2,036 (7)
Stage				
I Localized	1,022 (24)	299 (23)	6,743 (27)	8,064 (26)
ll Regional	686 (16)	196 (15)	4,014 (16)	4,896 (16)
Ill Distant	914 (21)	311 (23)	5,260 (21)	6,485 (21)
Unknown	1,654 (39)	525 (39)	9,097 (36)	11,276 (37)
Years from cardiovascular disease to prostate cancer				
0-1	1,278 (30)	375 (28)	-	-
2-10	2,998 (70)	956 (72)	-	-
Period of prostate cancer diagnosis				
1997-2000	942 (22)	250 (19)	6,093 (24)	7,285 (24)
2001-2004	1,378 (32)	445 (33)	7,721 (31)	9,544 (31)
2005-2008	1,956 (46)	636 (48)	11,300 (45)	13,892 (45)
Total number of deaths during study period	2,067	732	11,052	13,851
Prostate cancer	1,204	418	7,530	9,152
Cardiovascular diseases	450	155	1,213	1,818
Other causes	413	159	2,309	2,881
Number of years at risk (years from diagnosis to death or December 31^st ^2008)	11,564	3,201	74,922	89,687
Cause of death per 1,000 years at risk				
Prostate cancer	104	131	101	102
Cardiovascular diseases	39	48	16	20
Other causes	36	50	31	32

*Level of Charlson Comorbidity index score (not including IHD, stroke, and prostate cancer) IHD = ischemic heart disease

### Survival

**Figure 1:**
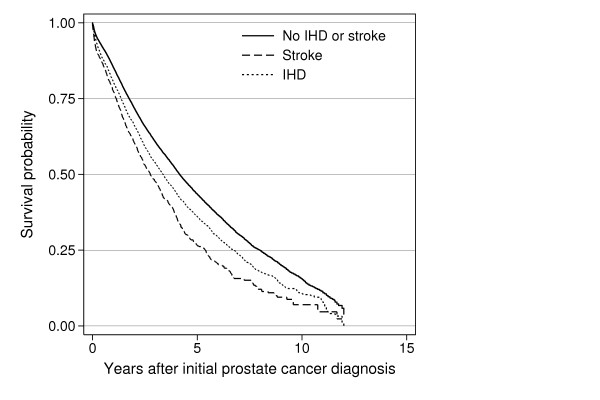
**Kaplan-Meier survival curves**. Kaplan-Meier curves showing survival probability of 30,721 prostate cancer patients by preexisting ischemic heart disease (IHD) or stroke status.

Figure 1 shows crude survival in the three cohorts of prostate cancer patients. Overall 1- and 5-year survival for the entire cohort was 84.4% (95% CI, 84.0-84.8) and 41.7% (95% CI, 41.0-42.4), respectively. Crude 1- and 5-year survival was lower for patients with preexisting IHD or stroke compared with those without IHD or stroke. Table 3 shows survival estimates for patient subgroups.

**Table 3:** Cumulative 1- and 5 year survival and crude and adjusted hazard ratios of entire cohort

Variables	No. of patients, n (%)	1-year cumulative survival	5-year cumulative survival	HR (95% CI)	
				
				Crude	Adjusted
Overall	30,721	84.4%	41.7%		
No IHD or stroke	25,114 (82)	85.4%	43.5%	1.0 (reference)	1.0 (reference)
IHD	4,276 (14)	80.5%	36.1%	1.25 (1.20-1.31)	1.05 (1.00-1.10)
Stroke	1,331 (4)	77.6%	26.5%	1.57 (1.46-1.69)	1.20 (1.12-1.30)
Stratified by age at prostate cancer diagnosis					
< 60 year					
No IHD or stroke	2,504	93.1%	62.3%	1.0 (reference)	1.0 (reference)
IHD	173	95.2%	50.1%	1.19 (0.90-1.57)	0.97 (0.73-1.29)
Stroke	53	90.6%	55.7%	1.28 (0.82-1.99)	1.18 (0.74-1.88)
60-69 year					
No IHD or stroke	8,202	92.3%	59.4%	1.0 (reference)	1.0 (reference)
IHD	1,082	91.8%	56.3%	1.08 (0.96-1.22)	0.94 (0.84-1.06)
Stroke	284	89.3%	44.8%	1.51 (1.23-1.85)	1.19 (0.97-1.46)
≥ 70 year					
No IHD or stroke	14,408	80.3%	33.2%	1.0 (reference)	1.0 (reference)
IHD	3,021	75.7%	28.8%	1.16 (1.10-1.23)	1.06 (1.00-1.12)
Stroke	994	73.6%	20.7%	1.38 (1.27-1.50)	1.20 (1.10-1.30)

Cumulative 1- and 5 year survival and crude and adjusted hazard ratios (HRs) of all cause mortality in 30,721 prostate cancer patients by preexisting ischemic heart disease or stroke status. Overall and stratified by age group.

Adjusted for age, stage, calendar period, comorbidity (except prostate cancer, IHD, and stroke) IHD = ischemic heart disease

### Mortality

A total of 13,851 (45%) prostate cancer patients died during follow-up (mortality rate, 154 per 1,000 person-years). Of those 2,067 had preexisting IHD (mortality rate, 179 per 1,000 person-years) and 732 preexisting stroke (mortality rate, 229 per 1,000 person-years). Prostate cancer patients with a history of IHD or stroke were more likely to die from other causes than prostate cancer, than patients without such history (Table 2).

Compared with patients without preexisting IHD the crude HR for patients with preexisting IHD was 1.25 (95% CI 1.20-1.31). For patients with preexisting stroke, crude HR was 1.57 (95% CI 1.46-1.69). After adjusting for age, stage, calendar period, and other comorbidities, both HRs decreased substantially (IHD 1.05, 95% CI 1.00-1.10, and stroke 1.20, 95% CI 1.12-1.30; Table 3). For prostate cancer patients below 60 years of age, preexisting IHD was associated with a lower mortality compared with patients without IHD or stroke (adjusted HR of 0.97, 95% CI 0.73-1.29; Table 3). This pattern was similar for patients aged 60 to 70 years (adjusted HR of 0.94, 95% CI 0.84-1.06), whereas patients older than 70 years of age with preexisting IHD had increased mortality (Table 3). In all age groups, prostate cancer patients with preexisting stroke had increased mortality compared with those without IHD or stroke.

Preexisting IHD did not affect mortality in prostate cancer patients without any other comorbidities (adjusted HR 0.99, 95% CI 0.92-1.07; Table 4) whereas prostate cancer patients with preexisting stroke and no other comorbidity had 42% higher mortality compared with prostate cancer patients without any comorbidity (adjusted HR 1.42, 95% CI 1.25-1.62; Table 4). We found no substantial difference in mortality between men diagnosed with IHD or stroke within one year before prostate cancer diagnosis and those diagnosed 2-10 years before prostate cancer diagnosis (data not shown).

**Table 4:** Crude and adjusted hazard ratios of prostate cancer patients without any other comorbidities

Variables	Total cohort (%)	HR (95% CI)	
		
		Crude	Adjusted
Overall	20,597		
No IHD or stroke	18,148 (88)	1.0 (reference)	1.0 (reference)
IHD	1,982 (10)	1.03 (0.95-1.11)	0.99 (0.92-1.07)
Stroke	467 (2)	1.53 (1.35-1.74)	1.42 (1.25-1.62)

Crude and adjusted hazard ratios (HRs) of all cause mortality in 20,597 prostate cancer patients without any other comorbidities.

Adjusted for age, stage, and calendar period

IHD = ischemic heart disease

## Discussion

In this nationwide cohort study of more than 30,000 prostate cancer patients, we found that preexisting IHD had only minimal impact on overall mortality, whereas prostate cancer patients with preexisting stroke had higher mortality than those without IHD or stroke.

For patients below70 years of age we observed better survival for patients with preexisting IHD compared with those without IHD. There are several possible explanations of why prostate cancer patients with IHD had better survival than prostate cancer patients without IHD. Because of their cardiovascular disease, these patients were probably in closer contact with the health care system, and were more concerned about their health which could lead to prostate cancer diagnosis at an earlier stage. We found, however, a higher prevalence of localized tumours in patients without IHD or stroke in all three age groups (data not shown), arguing against such surveillance bias. Other possible explanations include the beneficial effect of potential lifestyle changes after cardiovascular disease diagnosis (e.g. smoking cessation, increased exercise, weight loss) and the use of secondary medical prophylaxis, such as statins, which might be associated with reduced risk of advanced prostate cancer [19-21]. Due to lack of information about subsequent medical treatment this intriguing relationship could not be further explored.

We did not find a similar effect in patients with preexisting stroke; in contrast, these patients had substantially higher mortality than patients without IHD or stroke. We observed the largest increase in mortality among patients with preexisting stroke without any other comorbidities.

Our study revealed a considerable increase in the prevalence of IHD or stroke among prostate cancer patients during the study period. This is consistent with the general increase in prevalence of comorbidity among Danish cancer patients over the last decade [22-25]. Possibly, patients may be more likely to have a PSA test now than earlier during follow-up visits for IHD or stroke, which has resulted in more patients with IHD or stroke to be diagnosed with prostate cancer in recent years.

We cannot exclude the possibility that IHD or stroke patients who are diagnosed with prostate cancer present a selected group of patients, since these patients have actually survived their cardiovascular disease. This may bias the estimates towards the null and thus partly explain why the difference in mortality between those with and those without a history of cardiovascular disease is not larger than observed in this study.

Choice of treatment of prostate cancer is determined by many factors such as age, D'Amico risk group (PSA, Gleason score, and TNM stage), but comorbidity will also influence which treatment a patient is offered. Previous research on this topic has shown that prostate cancer patients with comorbidities are often offered nonsurgical/conservative treatment [4,7-9].

In the present study we found that preexisting stroke increased mortality to a significantly higher degree than preexisting IHD, which suggest a potential importance of differentiating between various kinds of comorbidities. When decisions are made about radical treatment of prostate cancer the type of comorbidity could be relevant to consider and not just the overall burden of comorbidities as expressed in the Charlson score.

The major strengths of our study were its large size, the virtually complete follow-up, and the ten year observation period before prostate cancer diagnosis to identify hospital-diagnosed comorbidity for each prostate cancer patient. The validity of the recorded diagnoses is crucial for the reliability of our findings. The registration of prostate cancers is thought to be virtually complete since free health care is available to all residents in Denmark: guaranteeing free access to hospitals and essentially eliminates all private inpatient or outpatient treatment of prostate cancer [16]. The positive predictive value of ischemic and unspecified stroke in the National Patient Registry has previously been estimated to be above 80% [26], and similar positive predictive values have been found for IHD [27,28].

Study limitations include use of a modified version of the Charlson Comorbidity index to control for confounding by other comorbidities, and it is unlikely that the index controls for confounding as effectively as clinical data [29]. Unfortunately, our study did not include information on prognostic lifestyle factors such as smoking or obesity or clinical data such as Gleason score, PSA, and treatments for prostate cancer and cardiovascular diseases which may have further informed our estimates.

## Conclusions

Our results showed that preexisting IHD had minimal impact on mortality following prostate cancer, whereas mortality was 20% higher in prostate cancer patients with preexisting stroke compared to those without IHD or stroke and even 42% higher for prostate cancer patients with preexisting stroke and no other comorbidities. These results highlight the importance of differentiating between various comorbidities when decisions are made on prostate cancer treatment.

## Competing interests

The authors declare that they have no competing interests.

## Authors' contributions

CGJ conceived the study, acquired data, participated in the design and analysis of the study, performed the statistical analysis, and drafted the manuscript. MN conceived the study, participated in acquisition of data, in the design and analysis of the study, and helped to draft the manuscript. TEBJ participated in the design and analysis of the study, and helped to draft the manuscript. MS helped to perform the statistical analyses of the study, and to draft the manuscript. MB conceived the study, participated in the design and analysis of the study, and helped to draft the manuscript. All authors read and approved the final manuscript.

## Authors' information

CGJ is a medical doctor and 2^nd ^year PhD student, MN is PhD and associated professor at the department of clinical epidemiology, TEBJ is PhD, associated professor, and chief surgeon at the department of urology, MS is PhD and post doc at the department of clinical epidemiology, MB is DMSc and professor of urology at the department of urology.

## Pre-publication history

The pre-publication history for this paper can be accessed here:

http://www.biomedcentral.com/1471-2407/11/519/prepub
